# Family Aggregation of HTLV-1 Infection Associated with FAS -670A/G Polymorphism: A Case Report

**DOI:** 10.3389/fmicb.2017.02685

**Published:** 2018-01-15

**Authors:** Antonio C. R. Vallinoto, Bárbara B. Santana, Maria A. F. Queiroz, Andrea N. M. R. da Silva, Izaura M. V. Cayres-Vallinoto, Carlos A. da Costa, Maisa S. de Sousa, Ricardo Ishak

**Affiliations:** ^1^Laboratório de Virologia, Instituto de Ciências Biológicas, Universidade Federal do Pará, Belém, Brazil; ^2^Núcleo de Medicina Tropical, Universidade Federal do Pará, Belém, Brazil

**Keywords:** human T-lymphotropic virus 1, family cluster, FAS gene, MBL gene, genetic polymorphism

## Abstract

Human T-lymphotropic virus 1 (HTLV-1) infection has been associated with ATL and inflammatory diseases but remains a neglected health problem. HTLV-1 associated diseases were originally described as sporadic entities, but family aggregations have been reported. Viral, genetic, immunological and behavioral factors were used to explain family clusters, but until now a clear explanation remains uncertain. In the present study we report, for the first time, a family cluster of diseased persons presenting the infection across three generations associated with FAS -670A/G polymorphism.

## Introduction

HumanT-lymphotropic virus 1 (HTLV-1) is a human retrovirus that causes lifelong infection (Poiesz et al., 1981; Bangham and Matsuoka, 2017). Recent estimate suggests that there are 5–10 million infected persons worldwide but its geographical distribution is heterogeneous, with the infection occurring in Japan, Australo-Melanesia, Caribbean, North and South America, Europe, Central, South and West Africa (Gessain and Cassar, 2012).

Although the infection has been associated with inflammatory and lymphoproliferative diseases (Bangham and Matsuoka, 2017) it remains a neglected health problem and many questions remain unsolved. It is not known why most HTLV-1-infected persons remain asymptomatic and 10% of them develop clinical symptoms (Kaplan et al., 1990). The presence of the virus is not the only variable associated with disease development once most of people infected with HTLV-1 remain asymptomatic.

Human T-lymphotropic virus 1 (HTLV-1) associated diseases were originally described as sporadic entities, but family clusters have been long reported worldwide (Ishak et al., 1995; da Costa et al., 2013; Alvarez et al., 2016; Frutos et al., 2017). Members of the same family share transmission routes of infection, virus genotypes, host genetic background, and environmental factors that increase the risk for family aggregation of the virus infection and associated diseases.

The search for host immunogenetic markers, possibly associated with HTLV-1 infection and progression to disease was pursued by our research group in the Amazon region of Brazil and other groups elsewhere (Nishimura et al., 1991; Jeffery et al., 2000; Pontes et al., 2005; Alves et al., 2007; Vallinoto et al., 2012, 2015; de Sá et al., 2016; Rosado et al., 2017). Polymorphisms rs1800450 (allele B, codon 54) and rs1800682 (-670A/G) located in *MBL2* and *FAS* genes, respectively, were strongly associated with HTLV-1 infection. *MBL2^∗^B* allele was associated with higher proviral load, a viral biomarker of disease outcome. Similarly, FAS -670A/G single nucleotide polymorphism allele *^∗^G* was more frequent among HTLV-1 asymptomatic infected persons and allele *^∗^A* was more prevalent among those with clinical neurological symptoms diagnosed as HTLV-1 associated myelopathy/Tropical spastic paraparesis (HAM/TSP). The present study aimed to investigate if a family aggregation of HTLV-1 infection could be associated with *FAS* and *MBL2* gene polymorphisms.

## Case Report

The study was approved by the Research Ethics Committee of the University Hospital João de Barros Barreto from the Universidade Federal do Pará (process No. 2061/2005). Participants were fully informed about the research objectives, and those who agreed to participate signed an informed consent form.

**FIGURE 1:**
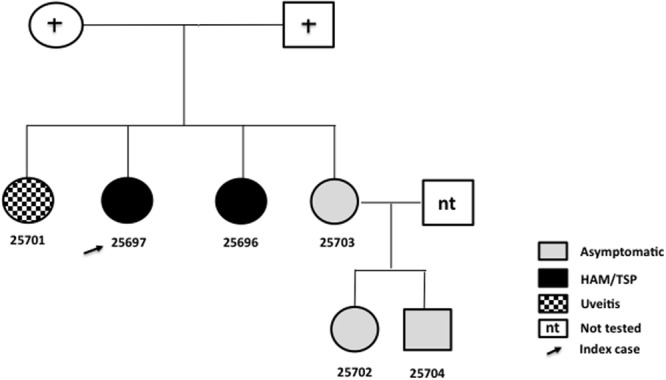
Heredogram showing a family aggregation of HTLV-1 infection and associated diseases.

**Figure 1** and **Table 1** describe a three generation family, from the Northern of Brazil (Amazon region), which included six members (five females and one male; index case #25697). HTLV-1 infection was investigated in the plasma and cells of blood samples. An enzyme immune assay was used for the detection of antibodies to HTLV-1/2 in the plasma. Positive samples were submitted to DNA extraction from PBMC using the purification kit GFX for genomic DNA (Amersham Pharmacia Biotech, Inc., United States) and assayed using nested PCR and qPCR to confirm the infection, as previously described (da Costa et al., 2013). *FAS* and *MBL2* gene polymorphisms were identified using a PCR method, as previously described (Pontes et al., 2005; Vallinoto et al., 2012).

**Table 1:** Demographic genetic and clinical information of the HTLV-1 infected family members investigated.

Subject	Sex	Age	Kinship	Generation (°)	MBL2	FAS -670	Clinic outcome	PVL
#25697^∗^	Female	61	–	2°	AA	AG	HAM/TSP	4529.8
#25696	Female	54	Sister	2°	AA	AA	HAM/TSP	2437.7
#25701	Female	65	Sister	2°	AB	AG	Uveitis	1215.0
#25703	Female	72	Sister	2°	AB	GG	Asymptomatic	1557.7
#25702	Female	49	Niece	3°	AA	GG	Asymptomatic	2652.3
#25704	Male	47	Nephew	3°	AB	GG	Asymptomatic	569.0

*^∗^Index case; PVL, proviral load (copies of DNA proviral/mm^*3*^).*

Infection with HTLV-1 subtype Cosmopolitan, subgroup Transcontinental, was confirmed in the six investigated members of the family, according to the homology to sequences deposited in the NCBI - National Center for Biotechnology Information website^1^.

Three members of the family were asymptomatic, two presented HAM/TSP and one with uveitis. Although the precise route of transmission was not known breastfeeding was the most probable route as two sisters reported to have been breastfed by the already deceased mother. One of the four sisters (#25703) was an asymptomatic married HTLV-1 carrier, who breastfed her two children and both were also asymptomatic HTLV-1 carriers.

*MBL2* gene polymorphism analysis showed the presence of AA wild genotype in three subjects. The allele *MBL2*^∗^B (rs1800450), carried by heterozygous genotype (AB), was observed in two sisters (second generation) and in one male of the third generation (**Figure 1**).

The investigation of FAS -670A/G polymorphism showed that five members of the family (83%) carried the allele *^∗^G*; three of them were asymptomatic homozygous GG and two were symptomatic heterozygous AG, one with a clinical diagnosis of uveitis and the other with HAM/TSP. One of the females with HAM/TSP was a carrier of AA genotype. The allele *^∗^G* was more frequent among the asymptomatic persons, but the allele *^∗^A*, was present among the three symptomatic sisters.

## Discussion

MBL is an important acute-phase serum protein involved in the innate immune response that can trigger complement activation (Wong et al., 1999), and an association between allele *^∗^B* in the exon 1 of the *MBL2* gene has been reported with the occurrence of immunodeficiency and chronic infectious diseases (Turner, 2003; Vallinoto et al., 2006). In our previous report, allele *^∗^B* was associated with higher proviral load, a biomarker of clinical progression for HTLV-1 associated diseases. One of the three family members carrying the allele *^∗^B* exhibited clinical symptoms of uveitis. The outcome of the other two asymptomatic carriers is still inconclusive and requires a follow up. However, two symptomatic HTLV-1 siblings (#25696 and #25697) carry the genotype AA, suggesting that there are other contributing factors for the progression of disease, apart from *MBL2* gene SNP.

FAS -670A/G polymorphism was investigated and the allele *^∗^G* was more frequent among the asymptomatic persons. Allele *^∗^A*, was present in three symptomatic sisters, supporting our previous report that showed higher frequency of allele *^∗^A* among HAM/TSP patients as compared to HTLV-1 asymptomatic persons (Vallinoto et al., 2012). The physical location of a SNP of FAS (-670A/G) favor its binding to the site of the signal transducer and activator of transcription (STAT1), which would be sufficient for the up and down regulation of *FAS* gene (Wang et al., 2016). Our previous report describes a strong association between this SNP and HTLV-1 infection and suggests that persons with FAS -670GG genotype may have a lower affinity to STAT1 binding as compared to infected persons carrying the FAS -670AA genotype. The presence of the SNP may lead to a decrease or increase of the apoptotic potential of the FAS receptor among patients carrying FAS -670GG and AA genotypes, respectively (Vallinoto et al., 2012). Farre et al. (2008) showed that the FAS promoter polymorphism is associated with the clinical manifestation and survival of HTLV-1 patients presenting adult T-cell leukemia – ATL. Among HAM/TSP patients, FAS levels correlated positively with lymphocyte activation, but the increased lymphocyte FAS expression was linked to decreased apoptosis and increased lymphoproliferation in cell culture and a strong increase in cellular FAS expression upon HAM/TSP progression (Menezes et al., 2017). Moreover, Rosado et al. (2017) found an association between the FAS -670 AA genotype and the higher proviral load among HAM/TSP patients.

It is important to point out that although familial aggregation of HTLV-1 infection and disease is not a recently described characteristic of the natural history of the virus, there are few attempts to characterize associated biomarkers, which favor the clustering of cases and define the outcome of the infection. FAS -670A/G polymorphism seems to be a reliable indicator to pursue involving larger numbers of other families with HTLV-1 aggregation. This would certainly help to assist toward prevention of the virus spread.

## Author Contributions

Conceived and designed the experiments: AV, MS, and RI; wrote the paper: AV, IC-V, and RI; assisted with editing the paper: MQ, AS, and IC-V; performed the experiments: BS, CC, and MQ; analyzed the data: AV, BS, RI, and MS; contributed reagents/materials/analysis tools: AV, MS, and RI.

## Conflict of Interest Statement

The authors declare that the research was conducted in the absence of any commercial or financial relationships that could be construed as a potential conflict of interest. At the time of acceptance, the handling Editor declared a shared affiliation, though no other collaboration, with the authors.

## Footnotes

1 https://www.ncbi.nlm.nih.gov
